# Tailored Approach to Evaluation and Management of Early Onset Neonatal Sepsis in a Safety-Net Teaching Hospital in Northeast Florida

**DOI:** 10.7759/cureus.45263

**Published:** 2023-09-14

**Authors:** Sfurti Nath, Rana Alissa, Samarth Shukla, Meng Li, Carmen Smotherman, Mark L Hudak

**Affiliations:** 1 Pediatrics/Neonatal Perinatal Medicine, University of Florida College of Medicine – Jacksonville, Jacksonville, USA; 2 Pediatrics, University of Florida College of Medicine – Jacksonville, Jacksonville, USA; 3 Neonatology, AdventHealth for Children, Orlando, USA; 4 Pediatrics, Pediatric First, Warner Robins, USA; 5 Pathology/Biostatistics, University of Florida College of Medicine – Jacksonville, Jacksonville, USA

**Keywords:** antibiotic stewardship, neonate, new born, sepsis risk calculator, early onset neonatal sepsis, early onset sepsis

## Abstract

Objective

Early onset neonatal sepsis (EONS) remains a significant cause of morbidity and mortality in newborns in the immediate postnatal period. High empiric antibiotic use in well-appearing infants with known risk factors for sepsis led the American Academy of Pediatrics (AAP) to revise its 2010 guidelines for the evaluation and management of EONS to avoid overuse of antibiotics. In this recent clinical report, the AAP provided a framework that outlined several evidence-based approaches for sepsis risk assessment in newborns that can be adopted by institutions based on local resources and structure. One of these approaches, the sepsis risk calculator (SRC) developed by Kaiser Permanente, has been widely validated for reducing unnecessary antibiotic exposure and blood work in infants suspected of having EONS. In order to determine the utility and safety of modifying our institution's protocol to the SRC, we implemented a two-phased approach to evaluate the use of SRC in our newborn nursery. Phase 1 utilized a retrospective review of cases with SRC superimposition. If results from Phase 1 were found to be favorable, Phase 2 initiated a trial of the SRC for a six-month period prior to complete implementation.

Methods

Phase 1 consisted of retrospectively applying the SRC to electronic medical records (EMR) of infants ≥ 35 weeks’ gestational age admitted to the newborn nursery with risk factors for EONS between June 2016 and May 2017. We compared actual antibiotic use as determined by the unit's EONS protocol for evaluation and management based on 2010 Centers for Disease Control and Prevention (CDC) and AAP guidelines to SRC-recommended antibiotic use. We used EMR to determine maternal and infant data, blood work results, and antibiotic usage as well as used daily progress notes by the clinical team to determine the clinical status of the infants retrospectively. Based on the projected reduction in blood work and antibiotics use with the retrospective superimposition of SRC on this cohort of infants and identification of our high-risk patient subset, we developed a novel, hybrid EONS protocol that we implemented and assessed throughout Phase 2, a six-month period from August 2018 to January 2019, as a prospective observational study.

Results

Phase 1 (SRC superimposition) demonstrated that the use of the SRC would have reduced empiric antibiotic use from 56% to 13% in the study cohort when compared with 2010 CDC/AAP guidelines. However, these same findings revealed use of the SRC would have resulted in delayed evaluation and initiation of antibiotics in 2 of 4 chorioamnionitis-exposed infants with positive blood cultures. During Phase 2 (n=302), with the implementation of our tailored approach (SRC implementation with additional blood culture in chorioamnionitis-exposed infants), 12 (4%) neonates received empiric antibiotic treatment compared to nine (3%) neonates who would have been treated per strict adherence to SRC recommendations. No neonate had culture-positive EONS. Continued use of 2010 CDC/AAP guidelines would have led to empiric antibiotic use in 38 (12.6%) infants in this cohort.

Conclusion

We developed a novel hybrid approach to the evaluation and management of neonates at increased risk of EONS by tailoring SRC recommendations to our safety-net population. Our stewardship effort achieved a safe and significant reduction in antibiotic usage compared to prior usage determined using CDC/AAP guidelines.

## Introduction

Early onset neonatal sepsis (EONS) remains a significant cause of morbidity and mortality in newborns in the immediate postnatal period despite a steady decline in its incidence over the past three decades [1]. EONS is defined as a positive blood or cerebrospinal fluid culture within the first 72 hours of life in infants in association with clinical signs consistent with sepsis. Collaborative efforts by the Centers of Disease Control and Prevention (CDC), the American Academy of Pediatrics (AAP) and the American Academy of Obstetrics and Gynecology (ACOG) directed at reducing maternal transmission of group B Streptococcus (GBS), one of the leading causes of EONS, as well as evidence-based evaluation and management (E&M) guidelines for at-risk infants have led to a reduction in incidence of EONS from 1.8/1000 live births in the mid-1990s to the current incidence rate of 0.3-0.8/1000 live births [1-3].

Robust surveillance of epidemiological and outcomes data has led to the subsequent revision of these E&M guidelines (in 2002 and again in 2010) [2,4]. These guidelines were based on the presence of validated perinatal risk factors for EONS such as prolonged rupture of maternal membranes, clinical chorioamnionitis, and inadequate intrapartum antibiotic prophylaxis for GBS as well as serial neonatal clinical exams with recommendations for blood work and/or empiric antibiotic treatment for infants identified to be at-risk for sepsis in the algorithm [2,3,5]. While useful with a high incidence of EONS, these guidelines continued to recommend blood work and empiric antibiotic treatment in a large number of at-risk infants screened by the 2010 CDC/AAP algorithm, even when the incidence of EONS was low and infants were clinically well-appearing and stable [6]. Additionally, the algorithm neither quantitated absolute risk nor provided any index of relative risk for sepsis resulting in a large percentage (15%-20%) of term and late preterm infants being evaluated for sepsis, with 5% to 8% receiving empirical antibiotics. Some studies estimated that for each episode of culture-positive EONS, as many as 1400 well-appearing infants received empiric antibiotics [7,8].

Several attempts to address these concerns of overtreatment in healthy, unaffected infants were dampened by the high risk of mortality in untreated or missed cases of EONS in this fragile population [7]. One innovation, Kaiser Permanente’s Sepsis Risk Calculator (SRC), showed promising results in estimating the risk for EONS in infants by using a multivariate analysis of perinatal risk factors and an infant's clinical assessment [8-10]. As part of their quality improvement project, the center reported a 50% reduction (from 5.0% to 2.6%) in the rate of empiric antibiotic administration among infants born at 14 hospitals with the use of the SRC [11]. This approach has been widely validated by other centers for reducing unnecessary antibiotic exposure and blood work in infants suspected of EONS. A second approach based on published literature, but less widely used due to resource limitation in most centers, advocated the use of serial, frequent physical examinations and initiation of antibiotics in infants who developed clinical signs consistent with sepsis [12,13].

As a result, the 2018 revision by the CDC/AAP of its 2010 guidelines provided a framework of several evidence-based approaches for sepsis risk assessment in newborns that can be adopted by institutions based on local resources and structure. Facilities can choose from one of the aforementioned approaches or continue their use of the 2010 CDC/AAP guidelines in consideration of population demographics and resources available to the mother-baby unit [9].

Several centers have subsequently modified their institution's approach to the evaluation and management of EONS based on these recommendations by either adopting one of the two approaches or modifying their existing approach within the framework of the evidence-based recommendations by CDC/AAP (2018) including recommendations from centers that reported safe implementation of the SRC with reductions of laboratory evaluations and antibiotic treatment of newborns [14-17]. However, the use of the original SRC also resulted in rare instances of missed or delayed treatment of infants with EONS. These instances led to the creation of expanded management options within the SRC (e.g., "vitals Q4hrs" and "consider antibiotics" options as part of SRC management recommendations) [18].

For this study, the 2010 guidelines formed the protocol of care for E&M of infants admitted to the newborn nursery (NBN) in a teaching hospital in Northeast (NE) Florida. In order to determine the utility and safety of modifying our institution’s current protocol (based on 2010 CDC/AAP guidelines) for EONS evaluation and management to the SRC, we implemented a two-phased approach to evaluate the SRC in our newborn nursery. Phase 1 utilized a retrospective review of cases with SRC superimposition. If results from Phase 1 were found to be favorable, results from Phase 1 would then be used to inform implementation of Phase 2 - a prospective trial of SRC in the NBN for a six-month period with the goal of safely reducing empiric antibiotic use in our high-risk population of infants ≥ 35 weeks’ gestational age admitted to the mother-baby unit.

## Materials and methods

Setting

We conducted our sequential, two-phase project in the NBN at the University of Florida (UF) Health Jacksonville. This facility is an academic, safety-net hospital in NE Florida that serves as the primary teaching site for the UF College of Medicine Pediatric residency program. This study was conducted as part of an internet-based, multi-site, national quality improvement (QI) collaborative organized by the Vermont Oxford Network (VON) called "Choosing Antibiotics Wisely" in which our institution was a participating site. It was approved by the University of Florida's Institutional Review Board (No. IRB201703244, IRB201702676).

Study design/method

Phase 1: Retrospective Superimposition of the SRC

Phase 1 was planned and conducted with the goal of evaluating if applying SRC to the electronic medical records (EMR) of infants, originally evaluated using our NBN's standard protocol of care for EONS (based on 2010 CDC/AAP guidelines), would decrease the rate of blood work and empiric antibiotic initiation and by how much. This was an important first step in our antibiotic stewardship effort before making any changes to our existing protocol. Criteria for inclusion to Phase 1 were, inborn infants admitted to the NBN, born at a gestational age of ≥ 35 weeks’ with one or more of the validated risk factors for EONS including positive or unknown maternal GBS status, chorioamnionitis, rupture of membranes > 18 hours, gestational age < 37 weeks, and who had a blood culture obtained for the evaluation of EONS. Out-born infants were excluded from Phase 1. 

We retrospectively identified from the EMR inborn infants admitted to the NBN over a period of 1 year (from June 2016 to May 2017) who met Phase 1 inclusion criteria. We ensured that infants in this cohort had one or more conditions that CDC/AAP had incorporated into their algorithm as risk factors for EONS (positive or unknown maternal GBS status, chorioamnionitis, rupture of membranes > 18 hours, gestational age < 37 weeks), and a blood culture had been sent for evaluation of EONS. We then applied the SRC for evaluation and management of EONS to the same infant records and compared the SRC recommendations with the recommendations that were originally used in the management of these infants (based on 2010 CDC/AAP guidelines). In doing so, we captured information on the actual and the SRC-recommended management including antibiotic usage, lab workup, or clinical observation, and identified all infants with positive blood cultures.

Data from Phase 1 was obtained and analyzed. An evidence-based consensus was obtained for the evaluation and management of EONS for Phase 2 after several deliberations to achieve buy-in from all stakeholders including neonatologists, pediatric hospitalists, and private pediatricians who admitted infants to our NBN as well as from nurses and leadership for Phase 2 protocol. This was followed by multiple educational sessions for all providers and nurses before implementation of Phase 2 (data gathering, analysis, consensus for Phase 2 protocol and education took place from June 2017 through July 2018). 

Phase 2: Prospective Application of Hybrid SRC

Results from Phase 1 informed Phase 2 to establish our new institutional EONS protocol. Given our hospital resources and our unique population characteristics, we made a detailed assessment of Phase 1 results, anticipating that we might either fully adopt the SRC or, alternatively, create a hybrid regimen for E&M that would better serve our high-risk population and address any safety concerns identified in Phase 1. We then carried out the observational portion of our study and prospectively evaluated an adapted SRC protocol over a six-month period (from August 2018 to January 2019). This adapted protocol was used for all infants that met inclusion criteria for Phase-2 which were, inborn infants admitted to the NBN, born at a gestational age of ≥ 35 weeks with one or more of the validated risk factors for EONS (positive or unknown maternal GBS status, chorioamnionitis, rupture of membranes > 18 hours, gestational age < 37 weeks). We excluded infants that were out-born from Phase 2. The recommendations for management obtained from the SRC were noted which included either routine observation, close clinical monitoring with more frequent clinical evaluations and vital sign monitoring, obtaining lab work without initiating antibiotics, or starting empiric antibiotics after obtaining lab work. Additionally, we were mindful to note observed changes in the recommendation for management by the SRC for infants that had a change in clinical status without validated risk factors for sepsis.

We used EPIC (the UF Health EMR) to extract maternal and infant demographic data and laboratory results pertaining to evaluation for sepsis. We completed an assessment of each infant’s clinical condition based on the assessment documented in the daily progress notes by the caregiver team. Data on each infant were entered into the SRC using Kaiser Permanente’s open-access web application (https://neonatalsepsiscalculator.kaiserpermanente.org/). All data obtained were stored in the pediatric department’s double password-protected research drive with access granted only to the study team members.

Statistical analysis

Descriptive statistics reported for this study include frequencies and percentages (categorical variables) and means, standard deviations, medians, and quartiles (continuous variables). We compared actual and SRC-recommended rates of antibiotic use using McNemar’s test for paired data. We compared continuous data using Wilcoxon rank sum tests and Fisher’s exact test for categorical data. In all cases, the level of significance was set at 0.05. All analyses were done in SAS® for Windows Version 9.4 (SAS Institute Inc., Cary, NC, USA).

## Results

Phase 1: retrospective study

Of 3215 total live births at UF Health from June 1, 2016, to May 31, 2017, 2850 infants were born at a gestational age ≥ 35 weeks. In accordance with the 2010 CDC/AAP guidelines, 175 infants admitted to the newborn nursery had a blood culture obtained and 98 (56%) of these were started on antibiotics at some time during the newborn nursery hospitalization. Characteristics of infants and mothers are summarized in Table 1 and Table 2, respectively.

**Table 1:** Demographic details and infant characteristics in study phase 1 and 2 APGAR: activity/muscle tone, pulse/heart rate, grimace (response to stimulation), appearance (color), and respiration/breathing; C-section: cesarean section; NICU: neonatal intensive care unit; SRC: sepsis risk calculator ^a^median (1st quartile; 3rd quartile) ^b^mean ± standard deviation ^c^count (percentage)

Characteristic	Phase 1 (n=175)	Phase 2 (n=302)
Gestational age, weeks^a^	37 (36,41)	39 (38,40)
Weight, grams^b^	3047 ± 572	3183 ± 487
APGAR – 1 min^b^	7 ± 2	7.71 ± 1.76
APGAR – 5 min^b^	8 ± 1	8.8 ± 0.67
Males^c^	95 (54)	157 (52)
C-section^c^	75 (43)	90 (30)
Resuscitation at birth^c^	37 (21)	44 (15)
Meconium at birth^c^	25 (14)	32 (11)
Mechanical ventilation^c^	22 (13)	9 (3)
Later transfer to NICU^c^	55 (31)	13 (4)
Antibiotics started at birth^c^	86 (49)	9 (3)
Antibiotics started after 12 hrs of birth^c^	13 (7)	3 (1)
SRC recommended empiric antibiotics at birth^c ^	23 (13)	9 (3)
SRC recommended labs/blood culture^c^	34 (19)	13 (4)

**Table 2:** Maternal characteristics in study, phase 1 and 2 GBS: group B Streptococcus; UTI: urinary tract infection; Deg C: degree centigrade ^a^median (1st quartile; 3rd quartile) ^b^mean ± standard deviation ^c^count (percentage)

Characteristic	Phase 1 (n=175)	Phase 2 (n=302)
Highest maternal temperature, Deg C^a^	37.1 (36.8,37.6)	37 (36.8,37.2)
Age, years^b^	27 ± 6	27 ± 6
Rupture of membranes, hours^b ^	10.49 ± 12.65	6.76 ± 8.33
Inadequate/no prenatal Care	33 (19)	45 (15)
GBS positive^c ^	55 (31)	221 (74)
GBS unknown^c ^	59 (34)	60 (20)
Chorioamnionitis^c^	49 (28)	30 (10)
UTI^c ^	5 (3)	12 (4)

On superimposition of the SRC to the portion of the infant cohort that had laboratory workup and/or were started on antibiotics in Phase 1, the SRC recommended laboratory evaluation and initiation of antibiotics in significantly fewer infants (19% vs. 100%, p < 0.001 and 13% vs. 56%, p < 0.001, respectively). In other words, the SRC would have recommended observation without empiric antibiotics in 87% of these infants. We found no difference in white blood cell (WBC) count between infants who received and who did not receive antibiotics (p = 0.94) as well as between those with positive and negative blood cultures (p = 0.56, Table 3). The immature:total (I:T) ratio was significantly different between infants who received vs. did not receive antibiotics (p < 0.001). There was no difference in I:T ratio between infants who had positive vs. negative blood cultures (p = 0.18, Table 3).

**Table 3:** Study phase 1, table showing WBC count and I:T ratio in treated and untreated infants and infants with positive and negative blood culture SRC: sepsis risk calculator; WBC: white blood cell; I:T ratio: immature:total ratio ^a ^p value, p significant if < 0.05 ^b ^p value for antibiotics per SRC ^c ^p value for positive blood culture

	Antibiotics per SRC yes (n=23)	Antibiotics per SRC no (n=152)	p^a,b ^	Blood culture positive (n=4)	Blood culture negative (n=171)	p^a,c^
WBC count	15.9 (12.5,19.3)	15.6 (12.3,19.0)	0.94	13.0 (10.5,20.0)	15.8 (12.4,19.0)	0.56
I:T ratio	0.19 (0.06,0.30)	0.06 (0.03,0.12)	<0.001	0.17 (0.13,0.48)	0.10 (0.04,0.22)	0.18

Four out of the 175 infants in Phase 1 had a positive blood culture. Each of these infants delivered in the setting of maternal chorioamnionitis and received antibiotics based on the 2010 AAP guidelines and NBN's protocol of care, but the SRC recommended antibiotic treatment in only two of the four infants. The first infant with SRC-recommended antibiotic treatment was full term, born to GBS negative mother with prolonged rupture of membranes (ROM) for 22 hours. Blood culture grew *Streptococcus anginosus*, which was judged to be a contaminant. The second infant was full term as well, born to a GBS-negative mother and the blood culture grew *Proteus*. However, SRC-recommended antibiotic treatment was not initiated for two well-appearing infants. The first of these infants was a late preterm twin of a set of Di-Di twins born at 36 weeks gestation. He developed transient bacteremia with GBS. The other twin remained asymptomatic with a negative blood culture. The second infant was full-term with prolonged ROM for 21 hours and developed *Escherichia coli* bacteremia. The common underlying risk factor in all these infants was exposure to maternal chorioamnionitis.

Phase 2: prospective use of a modified approach (hybrid SRC) to the evaluation and management of EONS

From June 2017 through July 2018, we abstracted and analyzed Phase 1 data to develop an evidence-based consensus for the evaluation and management of EONS in Phase 2. Based on the Phase 1 incidents where two well-appearing infants with positive blood cultures failed to elicit SRC recommendations to perform laboratory evaluation and treatment, our modified approach would require laboratory evaluation (blood culture) for all infants born to mothers with chorioamnionitis irrespective of SRC recommendations but would follow SRC recommendations for initiation of empiric antibiotics. SRC recommendations for evaluation and management were adopted for all other infants. During Phase 2, out of 1547 total live births between August 1, 2018, to January 31, 2019 (1314 infants born ≥35 weeks gestation), we identified 302 live-born infants who had one or more risk factors for EONS. Infant and maternal characteristics are summarized in Table 1 and Table 2, respectively. No neonates during this time period had EONS confirmed by a positive blood culture. Continued use of the 2010 CDC/AAP guidelines in these infants would have resulted in laboratory evaluations and empiric antibiotic therapy in 64 (21.2%) and 38 (12.6%) of infants, respectively. Our modified approach in Phase 2 reduced these numbers to 46 (15%) infants who had laboratory evaluation and 12 (4%) infants who received antibiotic therapy. Had we strictly complied with SRC recommendations, laboratory evaluations, and empiric antibiotics would have occurred in 13 (4%) and 9 (3%) of infants, respectively. Our higher rate of laboratory evaluation resulted from a blood culture prescription for any infant born to a mother with chorioamnionitis.

Regular six-month audits were performed to identify any cases of missed diagnosis of culture-positive EONS following a change in our practice and transition to the SRC, as part of the unit's routine quality assurance process. Two infants with culture-positive sepsis were identified by the SRC between February 2019 and January 2020 audits. Both infants were full term and blood culture was positive for *E. coli* and *Streptococcus viridians,* respectively. One infant was exposed to maternal chorioamnionitis and the other infant was delivered after prolonged ROM for 5 days. These infants had appropriate recommendations for obtaining blood cultures and starting empiric antibiotics at birth with the application of the SRC. No blood culture-positive infants were missed with strict adherence to the SRC which now forms the standard of care in our NBN for evaluation and management of EONS in infants ≥ 35 weeks gestation at birth.

## Discussion

Application of the 2010 CDC/AAP guidelines for the evaluation and management of EONS to the population of infants ≥ 35 weeks’ gestational age admitted to our safety-net newborn nursery has historically resulted in high rates of laboratory evaluation and antibiotic usage, due primarily to high maternal incidences of prolonged rupture of maternal membranes and/or clinical chorioamnionitis. Similar findings have also been reported at other centers [11,19]. The advent of Kaiser Permanente's SRC provided a multivariate population-based assessment of relative risk for EONS. Through this study, we aimed to refine a local approach to the evaluation and management of EONS while mindfully considering our high-risk population with the goal of reducing antibiotic utilization in a safe manner. A growing recognition of the adverse consequences of newborn exposure to systemic antibiotics [20-22] as well as immediately apparent consequences including delayed or impaired maternal-infant bonding, interference with breastfeeding, and excess NICU utilization and health care costs [23] contributed to the rationale for our efforts.

During Phase 1, we retrospectively compared the rate of antibiotic treatment of a population of infants who had full laboratory evaluation for sepsis per the 2010 CDC/AAP guideline with the rate that would have resulted from SRC recommendations. Analysis of this highest-risk group confirmed that adoption of the SRC would have reduced the rate of initiation of antibiotics from 56% to 13%, but that two well-appearing infants would have experienced delay in evaluation and treatment. As a result, we implemented a modified approach in Phase 2 in which we obtained blood cultures in all high-risk infants born to mothers with chorioamnionitis paired with SRC recommendations for initiation of empiric antibiotics and used SRC recommendations for evaluation and management in all other infants. Other centers that have revised their approach to the evaluation and management of EONS have adopted a similar phased approach in consideration of their populations until they acquired sufficient prospective data to allow robust insight into generalizability [17,24].

We tailored our modified approach to our unique patient cohort as well as to the resources of our hospital’s mother-baby unit. It aligned well with the CDC/AAP’s EONS policy update published in Dec 2018, which allowed centers three options for the evaluation and management of EONS [9]. While many centers have fully adopted the SRC, other centers have chosen to rely on frequent serial clinical examinations to identify early signs of sepsis and have reported no missed cases [25].

Each of the three approaches to initiation of treatment includes careful serial clinical assessments. Due to the development of clinical signs of sepsis after birth transition, 8% of infants in Phase 1 and 1% of infants in Phase 2 had blood cultures drawn after 12 hours of life with subsequent initiation of antibiotics. In deciding an approach to the evaluation and management of EONS, it is important to consider individual hospital resources, unique population factors, workflow constraints, and overall safety. It is equally important to be aware of each unit’s baseline EONS risk and to track outcome data to prevent oversights and safety mishaps.

Our modified approach did lead to a safe reduction in antibiotic utilization in Phase 2 to 4% from what would have been a rate of 12.6% had we strictly applied the 2010 CDC/AAP guideline. This outcome is in accord with what other centers have reported as decreased antibiotic usage for healthy late preterm and term infants following the adoption of the SRC [14-17,26,27].

Regardless of the approach adopted by a center, there will always be a possibility of missing infants with EONS. Some infants will develop EONS when a multivariate assessment defines low risk; other infants may develop EONS that is not treated promptly because of lack of timely recognition of clinical signs of sepsis. Each predictive model will be subject to error [28]. As emphasized by Benitz et al., a clinician should understand the strengths and limitations of any diagnostic tool [29]. These investigators further point to the methodological error in adapting the regression for application to a broader population (rather than the developmental cohort) and that some subsequent modifications may compromise the accuracy of quantitative predictions of the absolute risk of sepsis. The authors hence proposed that clinicians should not rely on the SRC to provide accurate estimates for the absolute risk of early-onset sepsis in newborn babies [29].

The major strengths of our study are the high-risk status of the study population and the sequential data-driven approach to achieving a consensus on a modified approach to the evaluation and management of EONS that was highly likely to reduce antibiotic utilization in a safe way. However, our study has some limitations as well. Phase 1 was a retrospective study that had the additional complexity of investigators having to make a post hoc inference about the clinical status of each infant based on the archived daily progress notes. During Phase 2, no infant developed EONS. Hence, we could not evaluate whether treatment was timely; however, the modified approach did guarantee that evaluation was accomplished in a timeframe that was equal to or faster than would have occurred solely based on the SRC.

Given that any prediction model is imperfect, it is clear that serial clinical examinations increase safety. Although reducing unnecessary use of antibiotics was the primary aim of our antibiotic stewardship efforts, we aimed to balance this against the safety concerns of our population. After each change in clinical practice, it is imperative that clinicians closely track and review outcomes and adverse events and adjust clinical protocols accordingly. Based on the outcomes and safety data from our study (Phase 1 and 2) and audit results, starting January 2020, our unit has successfully transitioned to completely adopting the SRC for evaluation and management of EONS in all infants admitted to our newborn nursery without obtaining additional blood culture in our high-risk, maternal chorioamnionitis exposed infants anymore and continue to perform six-month audits to evaluate for outcomes and safety data.

## Conclusions

We developed a novel hybrid approach to the E&M of neonates at increased risk of EONS by tailoring sepsis risk calculator recommendations to our safety-net population. Our stewardship efforts achieved a safe and significant reduction in antibiotic usage compared to our prior newborn nursery protocol based on recommendations from 2010 CDC/AAP guidelines. More studies are needed from different centers adopting any of the three approaches outlined in the 2018 CDC/AAP EONS policy update for early onset neonatal sepsis evaluation and management suited to their unique patient demographics and available hospital recourses. Regardless of the approach adopted, frequent audits to monitor outcomes and adjust clinical protocols accordingly are an essential part of any change in clinical practice.
